# Effect of TiO_2_ on the Gas Sensing Features of TiO_2_/PANi Nanocomposites

**DOI:** 10.3390/s110201924

**Published:** 2011-02-01

**Authors:** Duong Ngoc Huyen, Nguyen Trong Tung, Nguyen Duc Thien, Le Hai Thanh

**Affiliations:** Institute of Engineering Physics, Hanoi University of Science and Technology, No.1, Daicoviet, Hanoi, Vietnam; E-Mails: trongtung227@yahoo.com (N.T.T.); ducthien_ly@yahoo.com (N.D.T.); haithanh@mail.hut.edu.vn (L.H.T.)

**Keywords:** nanocomposites, TiO_2_, PANi, gas sensor

## Abstract

A nanocomposite of titanium dioxide (TiO_2_) and polyaniline (PANi) was synthesized by *in-situ* chemical polymerization using aniline (ANi) monomer and TiCl_4_ as precursors. SEM pictures show that the nanocomposite was created in the form of long PANi chains decorated with TiO_2_ nanoparticles. FTIR, Raman and UV-Vis spectra reveal that the PANi component undergoes an electronic structure modification as a result of the TiO_2_ and PANi interaction. The electrical resistor of the nanocomposite is highly sensitive to oxygen and NH_3_ gas, accounting for the physical adsorption of these gases. A nanocomposite with around 55% TiO_2_ shows an oxygen sensitivity of 600–700%, 20–25 times higher than that of neat PANi. The *n-p* contacts between TiO_2_ nanoparticles and PANi matrix give rise to variety of shallow donors and acceptor levels in the PANi band gap which enhance the physical adsorption of gas molecules.

## 1. Introduction

New materials with exceptional properties are urgently needed to satisfy the increasing demands coming from the electronic, photoelectronic and nanoelectronic industries [1–3]. The combination of organic and inorganic materials with different nature has been proposed as an effective approach to access complementary properties and synergetic effects [4–8]. Polyaniline (PANi), a typical semiconducting polymer with good environmental stability and usually considered as *p*-type semiconductor, has a variety of potential applications for lightweight battery electrodes, electro-magnetic shielding devices, anticorrosion coatings, and sensors [9]. The conductivity of PANi can be tuned by electrochemical redox, reversible acid/base doping/dedoping and it is then sensitive to environmental changes [10,11]. PANi is a type of material exhibiting sensing features at room temperature and convenient operation and therefore is an attractive prospect for the development of a variety of gas sensors. The properties of PANi, including its sensing characteristics, are found to be modified by dopants or by the interface interaction in a composite [12–14].

Titanium dioxide (TiO_2_), a typical oxide metal and *n*-type semiconductor exhibits interesting photocatalytic and electronic properties which has opened many promising applications in photo-voltaics, photocatalysis, photo-electrochromics and sensors [15–17]. TiO_2_, especially in the anatase form, can oxidize organic materials directly due to its strong oxidative activity, therefore the presence of TiO_2_ in the PANi matrix could cause some modifications and bring about new interesting properties, including sensing features. Based on these assumptions, an attempt was carried out to investigate the effect of TiO_2_ on the gas sensing characteristics of a TiO_2_/PANi nanocomposite synthesized by *in-situ* chemical polymerization.

## 2. Experimental Section

Aniline 99.5% (ANi), TiCl_4_ (Aldrich) and ammonium persulphate (APS, Kanto Chemical Co. Inc.) were used as starting materials to synthesize TiO_2_/PANi nanocomposites. All the materials were used as received. The procedure was carried out as follows: a solution of 40 mM TiCl_4_ was heated to 70–80 °C for 2 hours to make a sol of TiO_2_. The pH of the resulting solution after cooling down to room temperature was around 1.0 due to the formation of HCl. The TiO_2_ sol was then treated with a solution of 0.1 M aniline and 1.0 M HCl with different volumetric ratios. Then, a solution of 0.1 M ammonium persulphate with 1.0 M HCl used as an oxidant was added dropwise to the ANi and TiO_2_ sol mixture. The color of the solution gradually changed to blue, dark blue and dark green color, indicating the polymerization of PANi was occurring in the solution. After 2 hours the dark green deposited materials (TiO_2_/PANi nanocomposite) were filtered out, washed repeatedly with distilled water, rinsed in 1.0 M HCl solution and dried under vacuum. The nanocomposite morphology was characterized using FESEM (Hitachi-S4800) and TEM (Jeol) while the chemical and electronic structure was analyzed by FTIR, Raman spectroscopy (Nicolet 6700 NRX FT-Raman Module Spectrometer) and UV-Vis spectroscopy (Cary IE Varian). The gas sensitivity of TiO_2_/PANi composite was determined as its electrical resistor variation upon exposure to two kinds of gas with different chemical nature, namely oxygen O_2_ (an oxidizing agent) and ammonium NH_3_ (a reducing agent). The test TiO_2_/PANi layers were coated on interdigital Pt electrodes and the layer resistor changes were acquired and analyzed by a personal computer.

## 3. Results and Discussion

**Figure 1.**
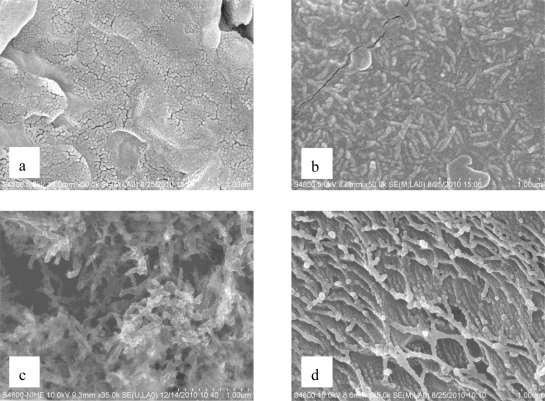
SEM images of TiO_2_/PANi nanocomposite with decreasing TiO_2_/PANi ratio: **(a)** 100 % TiO_2_; **(b)** 50% TiO_2_; **(c)** 10% TiO_2_; **(d)** 100% PANi.

SEM images show that the morphology of TiO_2_/PANi changes depended on the ANi concentration. Without ANi, TiO_2_ nanoparticles in the form of uniform granular with mean sizes around 20–25 nm are created (Figure 1a). When ANi is added, the resulting nanocomposite appears in the form of long PANi chains. The length of the chain increased with increasing ANi concentration (see Figures 1b, 1c, and 1d). The TiO_2_ nanoparticle size is significantly reduced in the presence of PANi.

**Figure 2.**
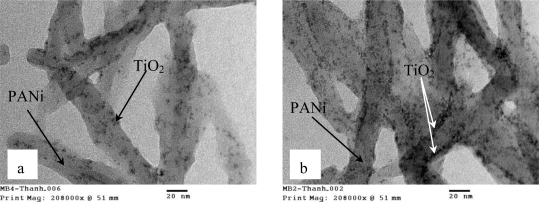
TEM images of TiO_2_/PANi nanocomposite show the appearance of TiO_2_ nanoparticles on surface of PANi: **(a)** 10 % TiO_2_; **(b)** 50%TiO_2_.

As can be seen from the TEM images in Figure 2, black TiO_2_ nanoparticles with sizes around 3–4 nm are randomly distributed in the PANi matrix and tend to line up along the PANi chains. Consecutive *p-n* junctions of TiO_2_ nanoparticles and PANi are formed, as a result a variety of shallow levels (both donor and acceptor) are created in the nanocomposite.

**Figure 3.**
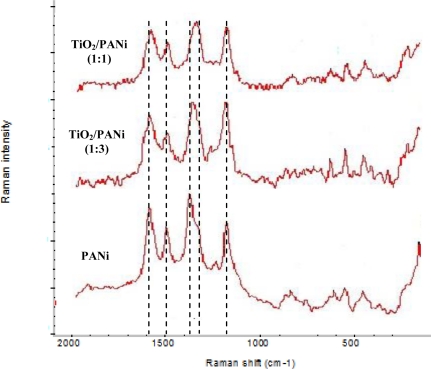
Raman spectra of PANi and TiO_2_/PANi nanocomposites.

Raman spectra recorded for the TiO_2_/PANi nanocomposites (see Figure 3) show the vibration modes characterizing of PANi emeradine and anatase TiO_2_. In the Raman spectrum of the anatase single crystal TiO_2_ the following modes can be assigned: ∼144 (E_g_), 197 (E_g_), 399 (B_1g_), 513 (A_1g_), 519 (B_1g_) and 639 cm^−1^ (E_g_). From the Raman assignment of the PANi vibration modes in Table 1, the band in the 1,490 cm^−1^ region is due to the C-N stretching vibration from benzenoid (B) ring while the band near 1,580 cm^−1^ is related to the C=N stretching from quinoid (Q) structure, the 1,140 cm^−1^ band is assigned to a vibration mode of the B-NH^+^=Q structure. It is an electronic-like band and is considered as a measure of the degree of delocalization of electrons. The three bands at 1,580, 1,490 and 1,140 cm^−1^ are slightly red shifted in TiO_2_/PANi nanocomposites indicating a slight effect of TiO_2_ on the bonds involved in these bands. The most striking point observed in Raman spectra is the red shift and increase in the intensity of the 1,338 cm^−1^, band which is assigned to the C-N^+^ stretching mode (semiquinoid form) of PANi in the nanocomposite. The 1,338 cm^−1^ band involves the polaron lattice and thus relates to the pristine conductance of the PANi.

As can be seen from Figure 3, the relatively higher intensities of the 1,140 and 1,338 cm^−1^ bands are evidence indicating that the PANi in TiO_2_/PANi nanocomposites has a higher degree of protonation than neat PANi. The TiO_2_-PANi interaction will loosen the chemical bonds and then TiO_2_ will play the role of counteranion and countercation along the polymer chain. From a physics point of view, when brought into contact with *p*-type semiconductor PANi, *n*-type semiconductor TiO_2_ may extracts some electrons from the valence band (HOMO) and adds some electrons to the conducting band (LUMO) of PANi and thus increases its conductance. The electronic structure of PANi then is modified by the interaction between TiO_2_ and PANi in the nanocomposite.

**Figure 4.**
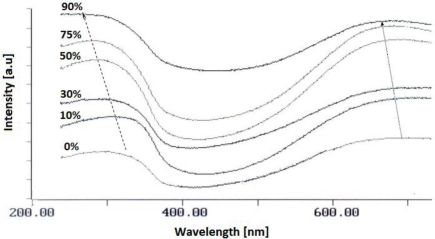
UV-Vis spectra of TiO_2_/PANi nanocomposites with different TiO_2_ contents.

The UV-Vis spectrum of TiO_2_/PANi nanocomposites (Figure 4) generally consists of two broad bands centered around 730–800 nm and 390–410 nm which originate from π → polaron, polaron → π^*^ and π → π^*^ transitions in the PANi emeradine salt. The positions of these bands are blue shifted depending on TiO_2_ concentration. The shift indicates a redistribution of polaron density in the band gap of PANi emeradine due to the impact of TiO_2_ nanoparticles.

**Figure 5.**
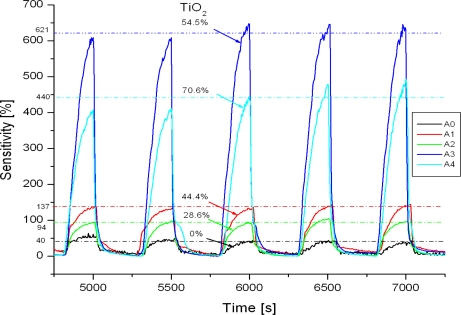
Oxygen sensitivity of TiO_2_/PANi nanocomposites with different TiO_2_ contents.

The conductivity of TiO_2_/PANi layers is found to be strongly affected by the environment. As can be seen from Figure 5 the resistance of TiO_2_/PANi layers changed with dry air pressure which was controlled by a vacuum pump. The reduction of air pressure causes a fast increase in the resistance of the TiO_2_/PANi layers and *vice versa*. The change in conductivity is accounted for by the interaction between oxygen (an electron acceptor) and PANi which loses more electrons in valence band then converts to be a *p*-type semiconducting species. The response and the recovery are fast, indicating the fact that the physical adsorption is dominant in the process. As can be seen from the plots, the sensitivity is strongly depending on TiO_2_/PANi ratio. Pure PANi exhibits a sensitivity of around 30–40%, while TiO_2_ shows no sensitivity. The highest sensitivity of around 650% is found in the composite containing around 55.0% of TiO_2_. In comparison to pure PANi, a 20–25 folds improvement in sensitivity is achieved. The appearance of a variety of *n-p* semiconductor contacts likely facilitates the formation of various gas molecular adsorption sites on the PANi surface (shallow levels). Oxygen, an electron acceptor, compensates for the sites gaining electrons on the PANi chain and this enhances the oxygen adsorption on the PANi surface.

**Figure 6.**
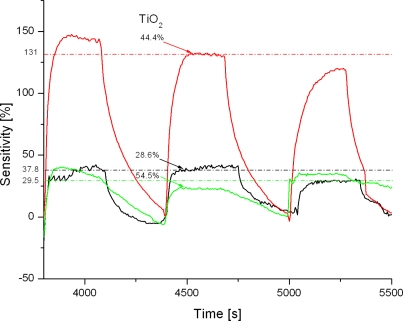
NH_3_ sensitivity of TiO_2_/PANi nanocomposites with different TiO_2_ contents.

The sensing behavior of TiO_2_/PANi nanocomposite upon exposure to NH_3_, a reducing agent acting as an electron donor, is different. The NH_3_ sensing profile of TiO_2_/PANi nanocomposite is shown in Figure 6. As can be seen from the figure, the resistance of TiO_2_/PANi layer increases as NH_3_ gas is injected. Upon interaction with NH_3_ some electrons are added into the valence band of the PANi *p*-type semiconductor, and as a result the conductance of PANi is decreased. The NH_3_ sensitivity of TiO_2_/PANi is high, but gradually declines with increasing cycles due to the saturation of NH_3_ adsorbed on the PANi surface. The nanocomposite with TiO_2_ concentration around 45.0 at % shows a peak in NH_3_ sensitivity. The effect of the *n*-type semiconducting TiO_2_ nanoparticles on the overall conducting properties of the nanocomposite is considered to be a reason, however the mechanism and the nature of the effect are still unclear.

## 4. Conclusions

TiO_2_/PANi nanocomposite synthesized by *in-situ* chemical polymerization formed long PANi chains with embedded TiO_2_ nanoparticles which tend to line up along the PANi chain with increasing TiO_2_ concentration. The length of the chain depends on the relative concentration of ANi monomer. FTIR, UV-Vis and Raman spectra show that the electronic structure of PANi is modified as TiO_2_ is brought into the nanocomposite. The conductivity of the nanocomposite layer increases upon exposure to O_2_ gas (an oxidizing agent) and decreases upon exposure to NH_3_ gas (a reducing agent). The process is reversible as a result from the physical adsorption and desorption processes which act as doping and dedoping on semiconducting PANi. Upon exposure to the oxygen in air the resistance of nanocomposite shows a 20–25 folds change in comparison to that of neat PANi. The *n-p* contacts between TiO_2_ nanoparticles and PANi matrix give rise to variety of shallow donors and acceptor levels increasing the physical adsorption sites for gas molecules thus enhancing the gas sensitivity.

## Figures and Tables

**Table 1.** Raman assignments of PANi bending and stretching bands.

**Frequencies (cm^−1^)**	**Assignments**
1120–1140	B-NH^+^=Q stretching
1230–1255	C-N stretching
1317–1338	C-N^+^ stretching
1470–1490	C-N stretching (B)
1515–1520	N-H bending
1580	C=N stretching (Q)
1600–1620	C-C stretching
